# Spelling in Deaf, Hard of Hearing and Hearing Children With Sign Language Knowledge

**DOI:** 10.3389/fpsyg.2019.02463

**Published:** 2019-11-12

**Authors:** Moa Gärdenfors, Victoria Johansson, Krister Schönström

**Affiliations:** ^1^Department of Linguistics, Faculty of Humanities, Stockholm University, Stockholm, Sweden; ^2^Center for Languages and Literature, The Joint Faculties of Humanities and Theology, Lund University, Lund, Sweden

**Keywords:** spelling, sign language, deaf, hard of hearing, CODA, writing processes, keystroke logging, spelling strategies

## Abstract

What do spelling errors look like in children with sign language knowledge but with variation in hearing background, and what strategies do these children rely on when they learn how to spell in written language? Earlier research suggests that the spelling of children with hearing loss is different, because of their lack of hearing, which requires them to rely on other strategies. In this study, we examine whether, and how, different variables such as hearing degree, sign language knowledge and bilingualism may affect the spelling strategies of children with Swedish sign language, *Svenskt teckenspråk*, (STS) knowledge, and whether these variables can be mirrored in these children’s spelling. The spelling process of nineteen children with STS knowledge (mean age: 10.9) with different hearing degrees, born into deaf families, is described and compared with a group of fourteen hearing children without STS knowledge (mean age: 10.9). Keystroke logging was used to investigate the participants’ writing process. The spelling behavior of the children was further analyzed and categorized into different spelling error categories. The results indicate that many children showed exceptionally few spelling errors compared to earlier studies, that may derive from their early exposure of STS, enabling them to use the fingerspelling strategy. All of the children also demonstrated similar typing skills. The deaf children showed a tendency to rely on a visual strategy during spelling, which may result in incorrect, but visually similar, words, i.e., a type of spelling errors not found in texts by hearing children with STS knowledge. The deaf children also showed direct transfer from STS in their spelling. It was found that hard-of-hearing children together with hearing children of deaf adults (CODAs), both with STS knowledge, used a sounding strategy, rather than a visual strategy. Overall, this study suggests that the ability to hear and to use sign language, together and respectively, play a significant role for the spelling patterns and spelling strategies used by the children with and without hearing loss.

## Introduction

This article concerns the writing skills of deaf and hard of hearing (henceforth, DHH) children, and focuses on the processes of writing and spelling. Having various degrees of hearing, or different language backgrounds may lead to different opportunities to develop a spelling ability, but the question remains whether and how those variables together, or separately, will mirror the spelling features of the deaf, and hard of hearing (henceforth, HoH) children.

To give an example, it is likely that when young, normally hearing children start learning how to spell, they will begin by basing the spelling on a sounding strategy, which in turn will cause typical misspelled words, with a close mapping of grapheme and phoneme (e.g., Nauclér, 1989). Bilinguals may exhibit cross-linguistic influence patterns in their language production, that is when structures in any language are influenced by their bilingual competence (see Figueredo, 2006, for a review). But we know next to nothing about the spelling patterns of children with hearing loss, who *at the same* time are bimodal bilinguals. A person is bimodal bilingual when their languages operates in two different modalities, for example using a sign language and a spoken language. Would such a context lead us to expect a pattern of transfer from sign language in the children’s spelling (i.e., will the children use a set of visual strategies for their spelling)? Is this case comparable to children who are hearing and bimodal bilinguals (i.e., those who use both sound-based and visual-based cues for their spelling)? Earlier research on the writing of DHH has mostly focused on deviations and errors (see Albertini and Schley, 2010 for an overview), and very few writing studies have included a sign language or a bilingual perspective over different language modalities *and* degrees of hearing.

Wengelin (2002) has reported that deaf adults with sign language knowledge misspelled fewer words compared to adults with reading and writing difficulties, and on the word-level this group barely demonstrated any doubling errors, which is an error type that is common in Swedish. Swedish spelling conventions require that many words include doubled consonants [e.g., “komma” (‘come’)]. To understand when a consonant should be doubled, and when not, requires both phonological and morphological knowledge, and spelling mistakes in this category are very common for all children in the targeted age group. Doubling errors include on the one hand errors when a consonant is erroneously doubled e.g., “villja” instead of “vilja” (‘will’), and on the other hand when the second consonant is erroneously missing [e.g., “tuga” instead of “tugga” (‘chew’)]. The deaf adults in Wengelin’s study, by contrast, showed more reversals, insertions, and morphological errors. The same study also showed that the deaf adults had a higher tendency to choose words which are visually similar to the target word, which resulted in a strategy that can be described as ‘spell as it looks’ – which was compared to a group of adults with reading and writing difficulties who spelled ‘as it sounds.’ Another finding was that the deaf adults showed a heterogeneous pattern, without common production problems, while the pattern was more homogenous in the adults with reading and writing difficulties. The strategies of the deaf group are thought to most likely be based on visual cues, where some patterns possibly could be derived from Swedish Sign Language (Svenskt Teckenspråk, henceforth, STS). Wengelin stresses that to find out with more certainty, an investigation of possible STS-influence, including what types of strategies or visual cues deaf people use to spell words, is needed.

The purpose of this study is therefore to perform a descriptive analysis of *whether* and *how* the spelling pattern is linked to children’s linguistic knowledge, not only of STS, but also of bilingualism, and hearing loss respectively, by looking at the spelling process *and* the final product through comparing children with and without STS knowledge, and with and without hearing loss. To our knowledge, this is the first study of its kind.

## Background

It is known that many DHH children face considerable challenges when learning to write. One factor behind these challenges is the absence of, or limited, hearing ability. Another factor is linked to the language acquisition background of the child. The literature often refers to the fact that more than 90% of deaf children are born into a hearing family without any contact with sign language (Mitchell and Karchmer, 2004). This may lead to a delayed start of language acquisition, and, in turn, the acquisition of written language can become a real challenge for the DHH children (Hall, 2017; Glickman and Hall, 2018). Nevertheless, studies show that deaf learners can become skilled readers and writers as well (Hoffmeister and Caldwell-Harris, 2014): a correlation between sign language knowledge and written language proficiency has been consistently reported (Strong and Prinz, 1997; Chamberlain and Mayberry, 2008; Freel et al., 2011; Kuntze et al., 2014). Previous research suggests that DHH children who are born into deaf families or, in exceptional cases, into families who started learning sign language early, may face a considerable advantage in their language development (see e.g., Svartholm, 2010 for an overview). Other studies have also shown that children with cochlear implant (henceforth, CI) with sign language knowledge outperform their DHH-peers born into hearing families without sign language knowledge in almost all intelligence tests (Amraei et al., 2017), in their speech and auditory development (Hassanzadeh, 2012), and showed comparable English scores with their hearing peers with sign language knowledge (Davidson et al., 2014), due to early first language acquisition. By contrast, some researchers have analyzed written outcomes for the deaf using the theoretical framework of Second Language Acquisition, arriving at the conclusion that deaf children exhibit grammatical structures similar to those of hearing second language learners in written Swedish (e.g., Svartholm, 2008; Schönström, 2014).

However, due to the variation in the DHH children’s different language (and hearing) backgrounds, it is difficult to arrive at general conclusions, as the relation between (or effect of) language experience (spoken/signed) and use, versus language proficiency and acquisition background (L1 or L2) remains understudied (cf. Hirshorn et al., 2015).

### Sounding Strategies in Spelling

A great deal of the research on literacy concerns phonological awareness. The first stage of developing literacy (in hearing children) is the development of phonological awareness that is, the knowledge of sounds, how sounds can be categorized into phonemes, and how sounds build words. An established phonological awareness has been proposed to constitute an essential foundation for reading, writing and spelling development (Nation and Hulme, 1997). According to the Swedish curriculum for the compulsory school, hearing students in grade three are assessed on their understanding of the relation between graphemes and sounds, but also the spelling rules for regular words, their mastering of the structure of Swedish, and their use of capital letters, question marks, exclamation marks and other punctuation. It can thus be expected that the fundaments of spelling are established for Swedish children when they begin 4th grade, which in Sweden means children of around 10 years of age (Skolverket, 2018).

An overreliance on phonological strategies as the foundation for spelling in hearing children can cause more spelling errors, since other factors (e.g., morphology) influence the orthographic rules (Frith, 1985), for instance Swedish orthography emphasizes doubling errors and letter substitutions (with recurrent examples from Bäck, 2011; Gärdenfors, 2016; Raatikainen, 2018).

From this it follows that normally hearing children have an advantage compared to the DHH, since their ability to hear helps in developing spoken language phonological awareness. When it comes to deaf readers and phonological awareness, Mayberry et al. (2011) conducted a meta-analysis of phonological awareness and reading skills, arriving at the conclusion that phonological awareness as a factor for deaf (and hearing) readers’ reading skills is overstated. Instead they found that language ability is a stronger predictor of reading achievement among deaf children. It should also be noted that DHH children can develop phonological awareness in sign language. Research has found positive correlations between signed phonological awareness and literacy skills. Profoundly deaf children can decode phonological information in sign language based on global characteristics from written words, fingerspelling or lipreading. Their solutions to code whole-words may therefore result in different misspelled words such as omissions (e.g., writing “orng*”* for ‘orange’), or letter reversals (e.g., writing “sorpt*”* for ‘sport’). Omissions, insertions and consonant errors have also been found in texts written by deaf children with sign language knowledge. The high number of consonant errors was explained as a consequence of lipreading, since the vowels are more distinct compared to the consonants (Sutcliffe et al., 1999). However, words will be easier to spell if they follow regular spelling patterns and children’s ability to decode spellings seems to emerge by second grade. DHH children with residual hearing (i.e., HoH and children with CI) have, however, been shown to be more sensitive to phonological information compared to their deaf peers with sign language knowledge (Marschark, 2009).

Some of those children seem to show certain similar misspelled word patterns, as has previously been reported in hearing children. Studies on children with CI or hearing aids have reported that the children’s access to sound enables many of them to use sounding strategies while spelling, causing “plausible” spelling errors (spelling errors based on sounds) (e.g., Geers and Hayes, 2011; Gärdenfors, 2016). Interestingly, Geers and Hayes (2011) further reported that CI-users using sign language in addition to the oral language made more errors that were not plausible. This compared to CI-users who only used spoken language. However, this group still faced difficulties in spelling due to lack of phonological awareness.

In a study on American English, Straley et al. (2016) explored spelling in narrative texts from twenty children using CI. The children were between 8.9 and 12.7 years of age, and all had spoken language as their primary language. The study found that, on average, 14% of all words were misspelled in the narratives. The children demonstrated an ability to represent correct sounds in words, which nevertheless resulted in misspelled words, such as doubling errors and omissions. However, it was shown that their spellings were not always conventional, which Straley et al. (2016) demonstrate with examples such as omissions (“cash” instead of “crash”), insertions (“drivier” instead of “driver”) and doublings (“ticet” instead of “ticket”). In the last example, the child is able to represent each sound in the target word, but fails to express the conventional spelling of the/k/sound, using the ‘ck’ diagraph (i.e., a combination of two letters that represents one sound).

In yet another study, 69 DHH children (with CI and hearing aids) using spoken language, between the age of 10 and 11, were compared with children with dyslexia. They were provided with a test battery consisting of standardized assessments such as non-verbal intelligence, reading and spelling, speech and language skills. The authors found striking similarities in spelling, word reading and non-word reading in both DHH children and children with dyslexia, and in line with earlier studies, the DHH-children showed poor phonological awareness. The children with dyslexia had a larger vocabulary than the DHH-children, and vocabulary was shown to be a strong predictor for good literacy outcomes for the group of deaf children, but not for the group with children with dyslexia (Herman et al., 2019).

Taken together, DHH children often face difficulties in phonological awareness (e.g., Harris and Beech, 1998; Geers and Hayes, 2011; Harris and Terlektsi, 2011; Bowers et al., 2016), likely due to their hearing loss (Sterne and Goswami, 2000; Alamargot et al., 2007). The degree to which sign phonological awareness is transferred to spelling in writing, however, still remains unexplored. Our starting point in this study is that there might be differences depending on children’s hearing status as well as their language abilities and backgrounds (cf. Hirshorn et al., 2015).

### Spelling in Bilinguals

Swedish spelling research in bilinguals or second language learners on the word-level is limited, to our knowledge, to a handful of student essays, thus referring us to the international spelling literature. Figueredo (2006) reviewed twenty-seven English as a second language (ESL) spelling studies. The most apparent difference between ESL and monolinguals regarding spelling development is that ESL-learners have an additional language that may be mirrored in their English spellings – i.e., a pattern of cross-linguistic influence through L1 transfer. An assumption within the concept of transfer is that if the language patterns in both languages are similar, the transfer would be facilitating, but if it differs, it would cause an interference, i.e., a so-called negative transfer. An example of interference is that Chinese and Japanese do not have relative clauses, resulting in Chinese and Japanese speakers using fewer relative clauses in English compared with other L2 who have relative clauses in their languages (Benson, 2002). Figueredo (2006) reported that the more the ESL-learners acquired the spelling norms of the second language, the less they relied on their first language, and that they followed the same spelling development as monolinguals.

A large study of reading and spelling development in the first two grades of elementary school included 1,812 children who were native speakers of Dutch and 331 bilinguals from Mediterranean or from Dutch colonies. The results showed that the spelling in the L2 was less efficient, and the children lagged in their phoneme–grapheme knowledge as well as phonemic segmentation compared to the L1. This was explained as difficulties in phoneme distribution rules, and that the children using the L2 had not developed the same automaticity for complex orthographic patterns and phonemic mapping as their L1 peers (Verhoeven, 2000).

Another study compared the ESL-learners with deaf children (mean age 10.7), also taking sign language into consideration in order to discuss possible transfer patterns from sign language to written English. The study found that deaf children made more omissions, insertions, and consonant errors and that the ESL-children showed more vowel errors and substitutions. Many spelling patterns of the deaf were non-phonetic and differed from the errors of the ESL children, who were more phonologically aware than the deaf children (Sutcliffe et al., 1999). The authors also found influence from British Sign Language (BSL) in the spelling of the deaf children. One fifth of those misspelled words of the British deaf children represented the initial letters only, which was explained by a fingerspelling influence from BSL through many incorporations of initialized signs (i.e., signs with a handshape corresponding to the fingerspelling of the word in the written/oral language) (Sutcliffe et al., 1999).

However, this point is debatable since Brown and Cormier (2017) reported that initialized signs in BSL are rare compared to one-handed systems such as American Sign language (ASL), indicating that BSL should be less amenable to initialization. Nevertheless, initialized signs are more common in one-hand systems such as in ASL. Lepic (2015) reported that approximately 15% of conventional lexical signs in ASL are initialized. There is no published study on initialized signs in STS, nevertheless a search in the STS corpus (Svensk Teckenspråkskorpus, 2019) shows that 13% of the STS signs are initialized. Padden and Ramsey (2000) report that skilled deaf signers could take advantage of initialized signs by using them as clues and translate them into English words. But, a “(non)initialized sign” can also cause false clues. Bowers et al. (2016) examined spelling in deaf children with ASL knowledge, and found that initial handshapes from ASL influenced the English spelling, such as “vorival” instead of “funeral.” This influence comes from the fact that the corresponding sign of “funeral” is expressed with two “V’s” using both hands.

Another study reported that deaf children showed fewer function words and had a high repetitiveness of the same words. It was suggested that this was a result of the fact that the function words in ASL are limited compared to English, and consequently this was a form of transfer from ASL (Singleton and Newport, 2004). In yet another study, this time of Dutch sign language, deaf students were divided into two groups: low- or high proficiency signing groups. The high-proficiency signing group was found to omit more obligatory articles compared to the low-proficiency signing group. This was explained to be an artifact of Dutch sign language, since sign languages often lack obligatory articles (Van Beijsterveldt and van Hell, 2010).

A very limited number of studies describe the literacy development of CODAs. Some report a similar literacy development pattern for CODAs as for hearing children’s first language acquisition (e.g., Brackenbury, 2005); others show that their language is reminiscent of second language learners (Larsson, 2015).

### Swedish Research on Spelling

In Sweden, the most comprehensive study of the spelling of hearing children is Nauclér (1980), who provides a deeper insight into different kinds of misspelled words typical for hearing children, especially concerning doubling errors which often are challenging for younger children. Here, doublings errors are defined as when a misspelled word lacks “required doubling and non-required doubling” (Nauclér, 1980, p. 55). In Swedish schools, children are often told to use the strategy to “listen to how it sounds” to find out the spelling of a word. This reflects a common misunderstanding about Swedish spelling that many Swedish teachers share; in fact, the Swedish spelling conventions can, in many cases, be better described as based on long, short, stressed or unstressed vowels. Spellers can use this information to figure out if the following letters will consist of one or two consonants, since the length or stress of an underlying vowel will determine the following number of consonants. However, there are several exceptions violating this rule (Nauclér, 1989). Beyond the doubling errors, there are other spelling error categories, such as insertions, omissions, inversions, letter substitutions, errors in diacritic letters, confusions of similar words and influence from STS.

Wengelin (2002) was the first Swedish researcher who observed the writing process of DHH adults with help of a keystroke logging tool. Today, there is a handful of writing process studies in DHH, using keystroke logging tools (i.e., Asker-Árnason et al., 2010, 2012). Keystroke logging has advantages for research on spelling. If misspelled words are analyzed in the final version of the text (which is the most common way to analyze spelling), we miss the opportunity to study the writing process during which the words were written (Wengelin, 2002). In the final text, we know nothing about which words may have been deleted from the text, or which words that may have been revised. Neither can we know about spelling attempts or cognitive efforts (measured by, e.g., pauses before, within or after a word with a spelling error).

### Fingerspelling

There are slightly different views on the linguistic status of fingerspelling in the sign language linguistics literature. Nevertheless, it is a regularly used component in many sign languages, including STS, as fully lexical signs (Johnston and Schembri, 2010; Hodge and Johnston, 2014). Using a manual alphabet, is used to convey places, personal names, or other words for which there is no sign equivalent. Fingerspelling is expressed in representations of written words and enables connections between a sign language and written words (Bergman, 2012). Fingerspelling is learned naturally and early, and studies have shown that younger deaf children understand fingerspelling as soon as they start learning to communicate, that they perceive it as signs, and they are also able to show attempts to fingerspell themselves. However, their attempts will naturally be limited due to motoric reasons (Padden, 2005; Bergman, 2012). Padden (2005) describes how deaf children learn to fingerspell twice – as young children they will first identify fingerspelling as a sign but as they get older, they will learn that fingerspelling has further linguistic patterns, and that a handshape represents a letter.

Kelly (1995) and Humphries and MacDougall (1999) showed that deaf adults (teachers as well as parents) use fingerspelling and chaining considerably more during communication with their students, compared to hearing teachers. *Chaining* is when an adult pedagogically gives a sign and/or points out a printed word and fingerspells it again to establish a connection between the sign and its written word. The difference between deaf and hearing adults lies in the fact that the deaf adults themselves had the experience of learning to understand the meaning of fingerspelled words before they could recognize printed words. Haptonstall-Nykaza and Schick (2007) showed that deaf children of deaf parents showed better results from fingerspelling training compared to deaf children of hearing parents. The same authors compared two ways to acquire English vocabulary: by a signing condition and by a fingerspelling condition. The results showed that the deaf children did better in the fingerspelling condition, under which they could recognize and produce more English words. The authors suggest that the lexicalized fingerspelling method is an appropriate way to establish a phonological link to printed words. Padden and Ramsey (2000) found a strong relationship between fingerspelling and reading ability in deaf children, and those who were skilled readers demonstrated good ASL-skills. The good signers were also more able to write down English words that were fingerspelled to them.

### Lipreading and Mouth Actions

Many children as well as adults with residual hearing need lipreading as a support to understand spoken language. However, trying to teach profoundly deaf children or adults to lipread is described as “difficult” and “frustrating,” since vowels are often visually distinct, while consonants are not. Deaf children have been shown to make more consonant errors in their spelling during writing as a result of trying to lipread a “silent” spoken word with invisible consonants (Sutcliffe et al., 1999; Marschark, 2009).

STS, as well as other sign languages, contains mouth movements i.e., mouth actions too. In sign language research, two main mouth categories have been identified so far: mouthing and mouth gestures (Crasborn et al., 2008). Beyond the lexical mouthing (mouth action patterns based on spoken language), there are also mouth gestures that provide a sign with further adverbial meanings such as regularity and intensity (Bergman, 1982, 2012). For this study, “mouthing” is relevant, as the visual phonetic elements from words in spoken languages are expressed without voice and used simultaneously with a manual sign, for example the Swedish sign for “HUS” (‘HOUSE’), uses mouthing based on the Swedish word for the house i.e., “hus.” However, unlike the spoken language, mouthing in STS follows a strict pattern, that is reduced in comparison to spoken languages. An example is the Swedish word “medlem” (‘member’) which is reduced to “mem” while signing it (Bergman and Wallin, 2001; SOU, 2006:29). In our data, spelling errors based on such reduced mouthing have been found in deaf children. Two recurrent errors are “falska” and “börd” instead of the correct “flaska” and “bröd” (‘bottle’ and ‘bread’). The reason is that STS mouth movements are reduced to “fa” and “bö” (Gärdenfors, 2016). Also, since “falska” and “börd” are existing words in Swedish (‘false’ and ‘descent’), consisting of the same, but reversed letters, it may be challenging for deaf children to learn the difference.

### The Present Study

In this study, we aim to examine how the four background variables of the DHH children: *STS knowledge*, *hearing loss, deafness* (including hard-of-hearing children without use of spoken language) and *bilingual* experience together, and separately, contribute to children’s spelling skills. Connected to this aim, we discuss which strategies and patterns DHH children, especially children with STS knowledge, show and use in their spelling. In this investigation, we have carefully selected children with different linguistic and hearing backgrounds, based on the four studied variables above. The participants consist of 33 children with variation in their degrees of hearing, use of spoken language, and in their language backgrounds, as being monolinguals or bilinguals. Each participant is categorized as a monolingual, unimodal bilingual (bilingual in two spoken languages), bimodal bilingual, (bilingual in spoken Swedish and in STS) or a sign-print bilingual, (bilingual in Swedish sign language and in written Swedish). Our research questions are the following:

- What do the writing processes and the spelling patterns look like in children with different linguistic and hearing backgrounds?
- Are any of the following variables: STS knowledge, bilingualism, hearing loss or, deafness, mirrored in these children’s writing and spelling patterns? If so, in which group of children?
- Can we identify which strategy the children with STS knowledge use in order to spell?

## Materials and Methods

### Participants

For the present study, 33 children (23 girls, 10 boys) between 9.9 and 11.6 years were recruited. Of these, 19 were children with STS knowledge (mean age: 10.9 years) and 14 were children without STS knowledge (mean age: 10.9 years). Their background information is presented in Table 1. 19 participants were bimodal bilingual, mastering Swedish and STS consisting of five deaf children, four HoH children, four CI-users and six CODAs. No spoken or written Swedish tests were administered, however, the background questionnaire reported no writing or reading difficulties for the children. Out of the 19 children with STS knowledge, seven DHH-children attended a school class for the deaf and had not developed any spoken language, whereas the other children could communicate by speech and attended a public school (i.e., mainstreamed with hearing children) or a special school class for hard-of-hearing children in which spoken Swedish was the primary language. All CODAs attended a mainstreamed school.

**TABLE 1:** Thirty three children participated in this study.

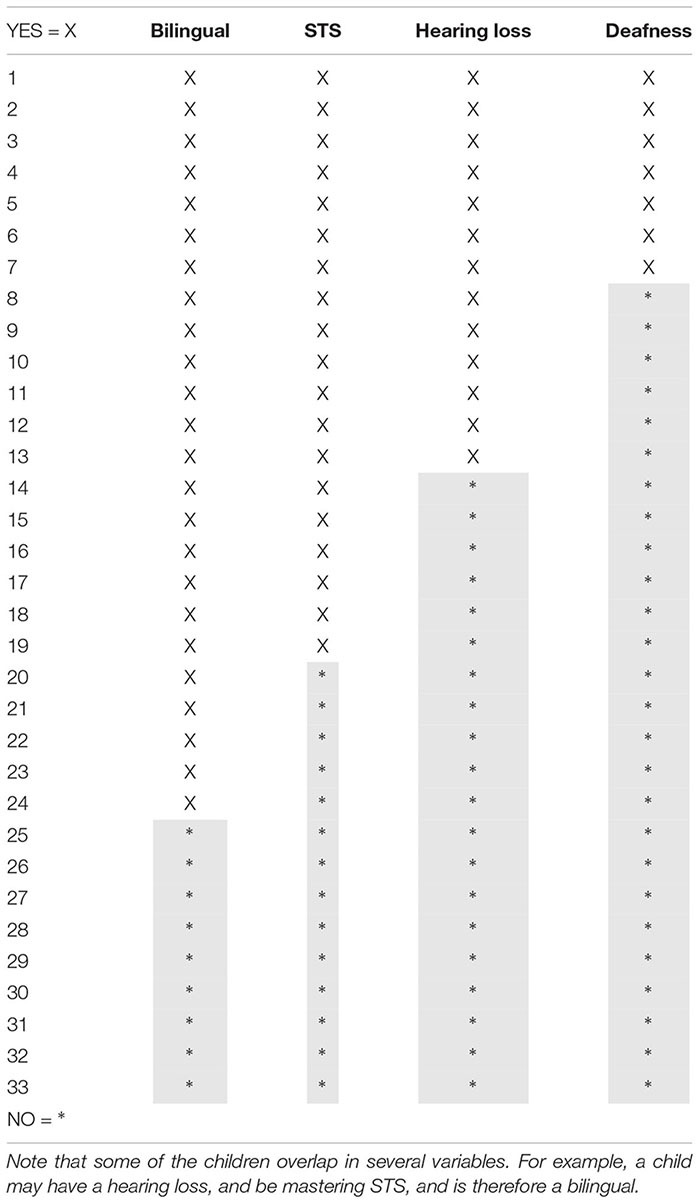

The remaining 14 children had normal hearing, and were without any knowledge of STS. This group consisted of five unimodal bilinguals and nine monolinguals. Beyond Swedish, the unimodal bilinguals communicated fluently in spoken Spanish, Danish, Thai, Dutch or Kurdish at home with their foreign-born parents. All of them attended a Swedish school and they were reported to master their two languages fairly equally. Unfortunately, we were not able to test their different languages, so our discussion about influence from other languages will be limited to possible influence from STS. The remaining nine participants were normally hearing monolinguals, mastering spoken and written Swedish.^1^

The inclusion criterion for DHH-children was that they should be born with hearing loss. Five children were profoundly deaf (<90 dB) and attended a class for deaf children. Four HoH children had a moderate to severe hearing loss without hearing aids (40–69 dB), and a mild to moderate hearing loss (25–54 dB) with hearing aids. However, two HoH-children have not developed spoken language and were therefore identified as deaf (Deaf HoH). All of the four CI-users were born profoundly deaf, and their first CI was implanted between the age of 9 months and 2 years and 2 months. Three of four CI-users have two implants, and with CI, their hearing was equal to a mild hearing loss (25–39 dB).

The inclusion criteria for the signing group was to be born into deaf families with STS knowledge, or into a family with parents who have started to learn and use STS early in the life of their child. Beyond the CODAs, 11 of 13 DHH-children had two deaf parents, and two children had two hearing parents, however, these parents had taken STS interpreter classes for several years (one of them is a certified STS interpreter) and are very skilled signers. In order to ensure the signing children’s STS-knowledge, we provided a STS-test, see the SignRepL2 section.

The scoring of SignRepL2 is based on a five-point scale, i.e., the maximal score for each STS sentence is 4 points (0–4) and the participants with STS as a first language tend to reach total mean score close to 4.0 on this test, while the children without any STS knowledge often are able to copy around the half of the manual signs only, due to the gesture content, but they leave out crucial linguistic parts of the signs, such as grammatical and non-manual functions. The test revealed that the children with STS-knowledge received a mean of 3.78 (SD: 0.19), and the children without STS-knowledge received a mean of 2.11 (SD: 0.20).

### Keystroke Logging

In order to capture the children’s writing processes, we used keystroke logging, a well-established method for investigating the writing process. In this case we chose to use ScriptLog, which is a program that documents everything the writer does with the keyboard or mouse during the writing session (Wengelin et al., 2019). This includes documenting revision processes, and pausing behavior. Through replaying or by studying a linear representation of the writing processes the researcher can understand more about the production of a text (Leijten and Van Waes, 2013). To the writer, ScriptLog looks like a simple word processor, with a start and stop button that can be administered by the researcher or the writer. In this version of the program, no spellcheck is included.

For the writing task, the children were provided with a two-page cartoon and were instructed to free-write a story from the cartoon on the computer. They were provided unlimited time, but their average writing time was 29.4 min. The output from ScriptLog consist of, on the one hand, the final text, i.e., the text as it is when the writer has finished writing, and on the other hand, generated information about their writing process, in the form of a linear text. The final text provides a starting point for analyzing linguistic features. Further, ScriptLog’s linear representations enable investigations of pauses and revisions, that took place during writing, but are not visible in the final texts.

### Writing Task

Children’s knowledge of the narrative genre is already established during pre-school years (Berman and Slobin, 1994), and we thus expected all the children in this study to have experience with, and basic knowledge of, the narrative genre. The stimulus for the written task consisted of a two-sided narrative cartoon about the *Pink Panther*. First, the use of picture-elicited narratives is a well-established method that has been used in earlier studies with Swedish DHH children (i.e., Schönström, 2010; Gärdenfors, 2016) and has provided robust outcomes of children’s written production, feasible for analysis. Second, as the scope of the cartoon’s content is delimited, the children are constrained to this in their writing, which leads to a delimited range of generated vocabulary output.

Further, this design enables us to compare and see how the children spell recurring words, and how they find other solutions such as synonyms and descriptions. The reason why we gave them unlimited time to finish the task was to eliminate the risk of them not showing their best ability if they got interrupted in the middle of the story. This choice was partly made based on the outcome of von Koss, Torkildsen et al. (2016), who provided 10 min writing time for their participants and found that assessment of the participants’ narrative competence was not accurate due to the shortness and incompleteness of their texts, caused by the time pressure.

Since the typing speed may be slower in younger children compared to older and more experienced writers, we expect that their low-level-processes, such as typing skills and spelling ability, will not be fully automatized yet, but that this will be evenly divided between the groups (cf. Berninger and Swanson, 1994; Wengelin, 2006). The average writing speed of the participants was 10.5 words per minute.

### SignRepL2

**FIGURE 1:**
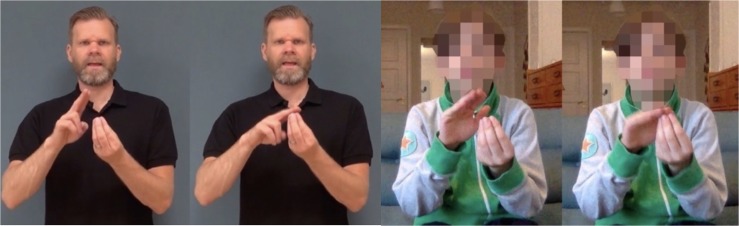
The first print screens from SignRepL2 represent the target sign for “ÄGG” [‘EGG’]. The second print screens represent a signing attempt of a participant who did not achieve full points because of incorrect hand shape and absence of mouth movement. Permission and written consents for using the print screens were obtained from the individuals and their parents, as well as the copyright holders of SignRepL2 (Schönström and Holmström, 2017).

In order to measure the participants’ STS proficiency, we used a STS repetitive test, called *SignRepL2* (Schönström and Holmström, 2017; Holmström, 2018). In the test the participants were shown fifty sentences, presented to them on a computer, and were asked to recall the sentences as presented, as exactly as possible during recording. The sentences increase in difficulty from simple single-sign items to three-sign sentences, see Figure 1. The 10-min test was originally developed for measuring L2 learners’ STS proficiency, but was used here for assessing the participants’ STS knowledge since there is no official standardized STS test for children available. This test has been tried out on 52 Swedish DHH children with STS as L1 or L2 by the developers of this test between 2016 and 2018 (Schönström et al. in preparation), and the measure of their STS results showed a valid difference between L1 signers and L2 signers. Based on this, and since no other tool is available, we expect that SignRepL2 should be suitable for the children of this study too. Testing STS knowledge is motivated by the fact that many writing studies on DHH children do not consider sign language in studying children’s reading or written proficiencies. Knowledge of the children’s STS proficiency is grounds for discussing possible cross-linguistic influence patterns between STS and writing.

### Procedure

The data collection was carried out in three steps. First, the children and parents were recruited through networks, schools or hospitals. After an appointment with a child was booked, the parents filled in a consent and background form about the child’s school, language use, and hearing background. The majority of the data collection took place in schools and hospitals. However, for practical reasons, some data was collected in homes in a quiet room. During the test sessions, the children received identical instructions from the first author, and they were informed that they could not ask for any help during the sessions. Every session started with the writing task and ended with the SignRepL2-test.

### Analysis of Writing Process and Spelling

An analysis of the writing process was the first step. Due to the automatic output from ScriptLog, a great deal of information from the writing process was retrieved: number of words, writing time, pause length, number of pauses, and pauses before, within and after words. For this study we used an *ad hoc* pause criterion of 1 s, which served the purpose of excluding the shortest pauses (which were more likely to be associated with motoric skills and finding a key), while including the longer pauses that could shed light on spelling processes. While we may have missed some relevant pauses, this pause criterion serves the purpose of the focus of the present study. Data further included measures of production rate, i.e., words per minute, and number of pauses per minute.

In addition, we manually identified all occurrences of misspelled words in the final text, and in the linear text, which included misspellings that were removed or corrected during the writing process. All spelling mistakes were sorted into eight different categories, see Table 2 for an overview. As a result, we could calculate every child’s spelling awareness, i.e., how likely it is that the child will detect and correct a spelling mistake. An example is that a child may misspell twenty times in total during the writing process and in the final text, but may only recognize five of them, and remove or correct them. Thus their spelling awareness will be 20% (5/20 = 20%). The higher the percentage spelling awareness is, the better the spelling. To our knowledge, this way of investigating spelling awareness by using keystroke logging is the first of its kind.

**TABLE 2:** Eight spelling error categories with descriptions are presented with examples of Swedish and English corresponding errors.

	**Description**	**Swedish examples**	**English examples**
Doubling errors	When a consonant is doubled or when the second consonant is missing (Nauclér, 1980)	Råta (Råtta) [‘rat’] Mussen (musen) [‘the mouse’] Äklig (äcklig) [‘disgusting’]	Faithfull, Ticet
Letter insertion	When an extra letter is inserted (Wengelin, 2002)	Taxsi (Taxi) [‘taxi’]	Priemary, Dierect
Letter omission	When a letter is missing (Wengelin, 2002)	Ijäl (ihjäl) [‘to death’]	Belive, Goverment
Inversions	When two letters change place (Wengelin, 2002)	Cylka (cykla) [‘bike’/’biking’]	Freind
Letter substitution	When an incorrect letter is replaced instead of the intended letter (Wengelin, 2002)	Sjönt (skönt) [‘pleasant’] Sengen (sängen) [‘the bed’]	Repitition, Definitaly
Errors in diacritic letters	Accurate in Swedish, when letters with dots are confused with other letters that look similar (Svartholm, 2006)	*A, Å, Ä* and *O, Ö* (Swedish) Äffar (*affär)* [‘store’]	
Confusion of similar words	When using a word that looks like another word (Andersson, 1994)	Fantastisk and faktisk [‘fantastic’ and ‘actually’]	Expect, except and desert, dessert
Influence from Sign Language	When a child shows any influence from STS, for example when a spelling error is influenced from STS reduced mouth movements (Bergman and Wallin, 2001) or from a handshape (Bowers et al., 2016)	Falska and börd (Flaska and bröd) [‘bottle’ and ‘bread’] Rätt instead of rädd (‘right’ and ‘scared’)	Sorpt instead of sport Vorival instead of funeral (the handshape for funeral is formed as a V in ASL)

The majority of the spelling analysis criteria derive from Wengelin (2002). First, existing words that were ungrammatical such as “Yesterday I have jump,” were not counted as misspelled words since the analysis excludes grammatical errors such as morphological errors. Another criterion is that if a word was used incorrectly in terms of meaning, for example “except” instead of the target word “expect,” this would be counted as a spelling error. Note, that a misspelled word may be included in two or more spelling error categories (Ejeman and Molloy, 1997). For example, the word “fela” (“fälla,” ‘trap’) belongs to the categories of doubling errors (when a consonant is doubled or when the second consonant is missing) and letter substitution (when an incorrect letter is replaced instead of the intended letter). Because of this, the concepts of misspelled words versus misspellings will be distinguished from each other, in order to avoid choosing a misspelled word belonging to a particular category by neglecting another. A misspelled word is taken to be the misspelled word itself, and the misspellings on the other hand are the number of misspelling categories counted in a particular misspelled word. The frequency of misspellings will, therefore, be greater than that of the misspelled words.

Writing texts on the computer, with the use of a keyboard, may result in writing errors unrelated to spelling skills. These “typos” typically occur when a writer presses an adjacent key instead of the intended one (e.g., ‘anf’ instead of ‘and’ on a QWERTY keyboard), or when a writer presses two keys in the wrong order (e.g., ‘adn’ instead of ‘and’). These so-called “typos” will generally not form any existing word, but may instead often violate the phonology of the language. The research literature that studies writing processes with keystroke logging has often observed that errors with typos are generally not associated with pauses (as an indication of increased cognitive load), and that typos are often immediately corrected. Wengelin (2002) compares typos to “counterparts in writing of articulatory errors in speaking” (p. 79). Research has shown that typos are common errors by children (and adults) who demonstrate no other spelling difficulties (Johansson, 2000). In the current study, we have chosen to exclude writing errors that can be categorized as “typos” from the spelling errors we investigate. The reason for this is that we are interested in describing the children’s spelling abilities, and not their general typing abilities, or abilities of error detecting.

### Statistical Analysis

In order to compare the results between the overlapping groups, we performed a regression analysis. As the means and SD were counted (see Table 3 for a result overview), the statistical analysis was fitted on all results, with help of the statistical program R, including number of words, writing length, words/minute, misspelled words in the final text, misspelled words in total, misspellings, spelling attempts, spelling awareness, pause length, pause/minute, number of pauses per minute, pauses before, within and after words, the STS-test and finally, the spelling error categories that can be found in Tables 4–6.

**TABLE 3:** The overview table displays the average and its SD in the overall categories: length measures, writing process, spelling error categories and STS-test in the variable no sign language, no bilingualism, no hearing loss and deafness.

**Participants (N)**	**19**		**14**		**24**		**9**		**13**		**20**		**7**		**26**	
**Variable**	**Sign language skills**		**No sign language skills**		**Bilingual**		**Monolingual**		**Hearing loss**		**Hearing**		**Deafness**		**Full or residual hearing**	
**Age**	**10.9**		**10.9**		**11.0**		**10.6**		**10.8**		**11.0**		**10.7**		**10.9**	
	**Mean**	***SD***	**Mean**	***SD***	**Mean**	***SD***	**Mean**	***SD***	**Mean**	***SD***	**Mean**	***SD***	**Mean**	***SD***	**Mean**	***SD***
**Writing Length**																
Number of words	268.5	114.2	296.9	109.0	284.8	121.9	269.4	81.1	221.8	86.5	318.8	110.6	175.7	64.3	308.8	104.6
Writing length in minutes	26.8	11.4	33.0	15.6	29.7	13.3	28.5	14.7	24.9	12.1	32.4	13.8	18.6	6.0	32.3	13.5
**Writing Process**																
Words per minute	10.7	3.8	10.3	4.5	10.2	3.5	11.3	5.3	10.0	4.5	10.8	3.8	10.4	4.7	10.5	4.0
Misspelled words in final text in%	4.5%	5	3.2%	2.7	4.2%	4.7	3.3%	2.9	4.6%	5.8	3.6%	3.0	3.3%	1.4	4.1%	4.8
Misspelled words in writing process and final text in%	6.5%	5.0	5.3%	4.6	6.1%	4.7	5.9%	5.3	6.7%	5.6	5.6%	4.3	5.6%	1.7	6.2%	5.4
Misspellings in final text in%	5.4%	5.4	3.4%	2.8	5.0%	5.0	3.4%	3.0	5.7%	6.1	3.8%	3.1	5.1%	2.2	4.4%	5.0
Number of spelling attempts	3.5	1.1	3.1	0.7	3.4	1.0	3.2	0.5	3.7	1.2	2.9	0.4	3.9	1.7	3.1	0.5
Spelling awareness in%	30.6%	26.1	40.6%	27.4	31.0%	27.5	43.1%	24.1	31.9%	28.0	36.3%	26.1	40.4%	26.0	34.0%	27.2
Pause length per text in%	61.5%	12.5	65.6%	8.9	62.9%	11.5	64.2%	10.9	62.4%	15.0	63.8%	8.1	61.2%	15.3	63.6%	10.1
Number of pauses per minute > 1 s	10.8	2.1	10.6	2.6	10.6	1.9	11.0	3.2	11.4	2.0	10.2	2.4	12.1	1.4	10.3	2.4
Pauses before words in%	29.1%	11.4	28.9%	10.5	29.5%	10	27.6%	12.4	29.5%	12.6	28.7%	1.0	33.4%	13.6	27.8%	10.0
Pauses within words in%	17.9%	9	19.5%	9.8	18.1%	8.6	19.7%	12.1	20.1%	9.8	17.2%	9.3	21.0%	7.7	17.9%	10.0
Pauses after words in%	7.2%	5.8	4.7%	2.3	6.7%	5.3	4.5%	2.0	9.2%	5.9	4.2%	2.3	9.5%	6.7	5.3%	3.8
**Spelling Error Categories**																
Doubling errors in%	2.3%	4.1	2.5%	2.3	2.5%	3.6	2.3%	2.6	2.2%	4.9	2.5%	2.2	0.5%	0.7	2.9	3.7
Insertions in%	0.3%	0.4	0.1%	0.2	0.3%	0.4	0.1%	0.2	0.4%	0.4	0.2%	0.4	0.3%	0.5	0.2	0.3
Omissions in%	1.1%	1.1	0.6%	0.8	0.9%	0.9	0.8%	0.9	1.3%	1.3	0.6%	0.7	1.5%	1.7	0.7	0.7
Inversions in%	0.3%	0.6	0.1%	0.2	0.3%	0.5	0.1%	0.2	0.4%	0.7	0.1%	0.2	0.5%	0.9	0.1	0.3
Letter substitutions in%	1.3%	1.6	1.7%	2.0	1.2%	1.4	2.3%	2.2	1.1%	1.4	1.7%	1.9	0.5%	0.6	1.7	1.8
Diacritic letters in%	0.4%	0.5	0.2%	0.5	0.3%	0.5	0.2%	0.4	0.3%	0.5	0.3%	0.5	0.4%	0.6	0.3	0.4
Confusion of similar words in%	0.7%	1.3	0.1%	0.2	0.6%	1.1	0.2%	0.2	1.0%	1.5	0.1%	0.2	1.8%	1.7	0.1	0.2
Influence from STS in%	0.4%	0.6	0.0%	0.0	0.3%	0.6	0.0%	0.0	0.5%	0.7	0.0%	0.0	1.1%	0.8	0.0	0.1
**STS Test**																
SignRepL2 (max 4.0)	3.8	0.19	2.1	0.20	3.4	0.69	2.1	0.18	3.8	0.19	2.6	0.78	3.9	0.03	2.8	0.83
																

**TABLE 4:** The overview table displays the results from the regression analysis on the investigated results: number of words, writing time, words per minute, pause length, pauses per minute, pauses before, within and after words based on no sign language, no bilingualism, no hearing loss and deafness.

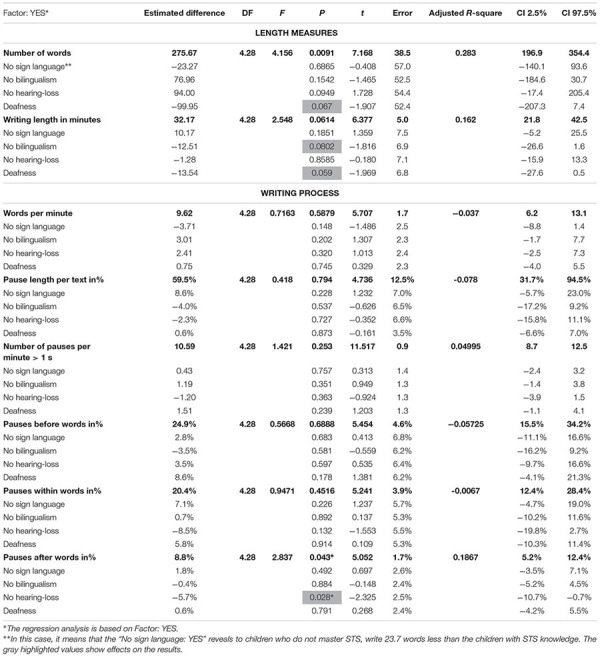

**TABLE 5:** The overview table displays the spelling results from the regression analysis on the investigated results: misspelled words in final text, misspelled words in total, misspellings, spelling attempts/text, spelling awareness and Sign-RepL2 based on the variables: no sign language, no bilingualism, no hearing loss and deafness.

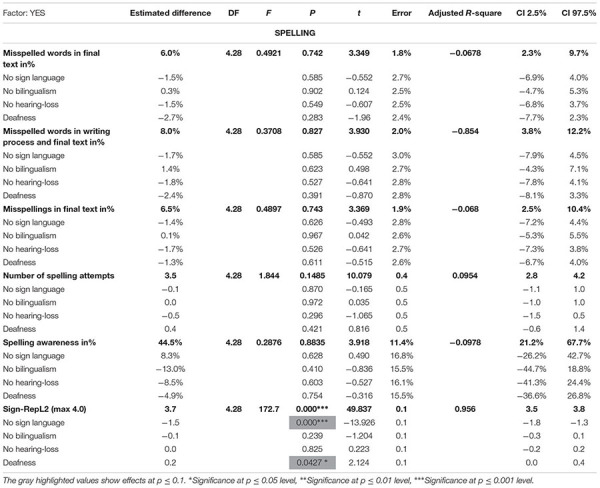

**TABLE 6:** The overview table displays the results from the regression analysis on the spelling errors based on the variables: no sign language, no bilingualism, no hearing loss and deafness.

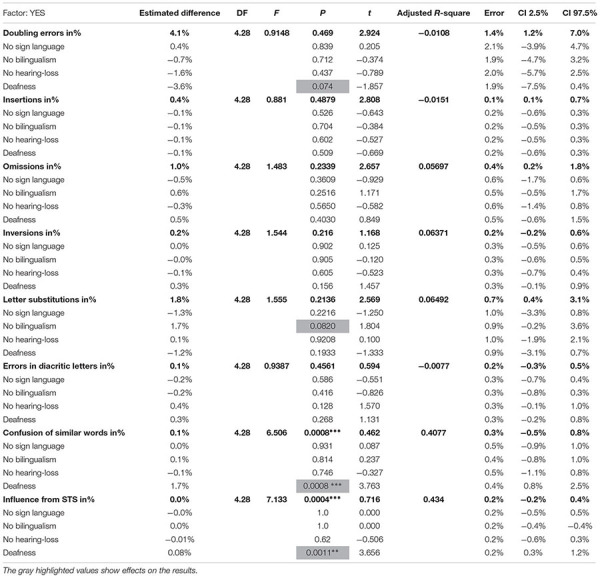

## Results

Table 3 provides an overview of the mean and SD, including length measures, writing processes, spelling errors and STS-test result. A regression analysis, based on Table 3, can be found in Tables 4–6. Tables 4, 5 show the statistical results on the length measures, writing process, spelling and the STS-test. Table 6 shows the statistical results on the spelling categories from Table 2.

### Overall Result of the Groups

In Table 3, the column to the left displays the overview result of the length measures, writing process and the spelling error categories divided by the variables: no sign language, no bilingualism, no hearing-loss and deafness displayed on the top of the table. The top column also displays number of participants in each group (N), their mean age, mean and SD of the results.

### Regression Analysis

In order to investigate the effects on the spellings, we performed a regression analysis on different writing and spelling measures, which are summarized and divided in Tables 4, 5. In these tables, the columns to the left display the results in length measures and writing process divided by the variables: no sign language, no bilingualism, no hearing loss and deafness. The next column displays the output from the regression analysis with the following: estimated difference, degree of freedom (DF), *F*-value, *P*-value, *t*-value, error, adjusted R-square and confidence intervals on a 2.5 and 97.5% level.

In this regression analysis, six effects (*p* ≤ 0.1) were found in Tables 4, 5, of which three were significant (*p* ≤ 0.05). The first effect was found on the number of words and deafness, *F*(4.28) = 4.156, *t* = −1.907, *p* = 0.067. The estimated word difference between the groups was −99.95 words, with a standard error of 52.4. The overall model fit was *F*(4.28) = 4.156, *t* = 7.168, *p* = 0.0091, *R*^2^ = 0.283. The second and third effects were found in writing time in minutes on the predictors no bilingualism (β = −12.51, *p* = 0.0802, with a standard error of 6.9) and deafness (β = −13.54, *p* = 0.059. with a standard error of 6.8). The overall model fit was *F*(4.28) = 2.548, *t* = 6.377, *p* = 0.0614, *R*^2^ = 0.162.

The first significant effect was found on pauses after words on the predictor, no hearing loss (β = −5.7%, *p* = 0.028^∗^, with a standard error of 2.5%), the overall regression model fit was *F*(4.28) = 2.837, *t* = 5.052, *p* = 0.043^∗^, *R*^2^ = 0.1867. Finally, two significant effects were found in SignRepL2, the STS test, (beta coefficient = −1.5, *p* < 0.000^∗∗∗^ with a standard error of 0.1), and in deafness: (beta coefficient = 0.2, *p* = 0.0427^∗^ with a standard error of 0.1). The overall model fit was *F*(4.28) = 172.7, *t* = 49.837, *p* < 0.000^∗∗∗^, *R*^2^ = 0.956.

In Table 6, the column to the left displays the investigated results on the *spelling error categories*, divided by the variables: no sign language, no bilingualism, no hearing-loss and deafness. The next column displays the output from the regression analysis with the following: estimated difference, degree of freedom (DF), *F*-value, *P*-value, *t*-value, error, adjusted *R*-square and confidence intervals on a 2.5 and 97.5% level.

Table 6 represents the second regression analysis that was fit on spelling error categories and four effects (*p* ≤ 0.1) were found in which two effects were significant (*p* ≤ 0.05). The first effect was found in the doubling error and deafness. *F*(4.28) = 0.9148, *t* = −1.857, *p* = 0.074. The estimated difference between the groups was −3.6%, with a standard error of 1.9%. The overall model fit was *F*(4.8) = 0.9148, *t* = 2.924, *p* = 0.469, *R*^2^ = −0.0108. The second effect was found in letter substitutions, with an effect on no bilingualism, *F*(4.8) = 1.555, *t* = 1.804, *p* = 0.0820. The estimated difference between the groups was 1.7%. The overall model fit was *F*(4.28) = 1.555, *t* = 2.569, *p* = 0.2136, *R*^2^ = 0.06492. The first significant effect was found in confusion of similar words with an effect on deafness. *F*(4.8) = 6.506, *t* = 3.763, *p* = 0.0008^∗∗∗^. The overall model fit was *F*(4.28) = 6.506, *t* = 0.462, *p* = 0.0008^∗∗∗^, *R*^2^ = 0.41. The last significant effect was found between influence from STS and deafness. *F*(4.28) = 7.133, *t* = 3.656, *p* = 0.0011^∗∗^. The estimated difference between the groups was 0.08%. The overall model was *F*(4.28) = 7.133, *t* = 0.716, *p* = 0.0004^∗∗∗^, *R*^2^ = 0.434.

### Interpretation of the Results

#### Writing Length

Effects (*p* ≤ 0.1) were found for number of words and writing length. The deaf children wrote on average 100 fewer words than the others. This can be explained by the well-documented fact that bilinguals in general have a smaller vocabulary in each language because of divided inputs from two languages (see Bialystok, 2009 for a review). However, there is yet another factor that explains *why* the deaf children on average wrote fewer words than the other bilinguals. Unlike the other bilinguals, they cannot take advantage of their hearing, so they are physically restricted in acquiring spoken Swedish by using their hearing. They cannot overhear conversations, on either TV or radio (Singleton, 2004). As a result, the constant input of Swedish through hearing is smaller than that of the other bilinguals. Taken together, a combination of shared input from two languages, and limited access to hearing, may be mirrored in a smaller vocabulary and shorter writing length.

#### Writing Process

Except for the low number of words and shorter writing time, all children, including deaf children, have developed similar typing skills. Between groups there were small differences regarding writing process measures such as words per minute, pause length, number of pauses, pauses before words, and pauses within words. Thus, all children in the study demonstrate similarly good transcription skills.

#### Spelling

The range of the misspelled words in percentage was not significant for the studied children, and in order to increase the validity, their results were compared with other Swedish spelling studies on normal-hearing and DHH children. The percentage of misspelled words for the hearing monolinguals of this study was surprisingly low, with an average of 3.3% in their final texts, while previous Swedish studies, including 67 children in similar age, showed an average of 8.5% misspelled words (based on studies by Bäck, 2011; Gärdenfors, 2016; Raatikainen, 2018). The teacher of the monolingual children in this study described them as an extraordinary class, so they have “set the bar high”. However, the monolinguals were not the only children with very few spelling errors – the CODAs, CI-users and HoH children showed very low percentages, with about half as many misspelled words compared to previous studies (Bäck, 2011; Gärdenfors, 2016; Raatikainen, 2018), except for the deaf group in which the number of misspelled words was equal to that found in Gärdenfors (2016). We have unfortunately not been able to find any comparable Swedish study on unimodal bilingual children.

One reason why the spelling errors in the children with STS knowledge were fewer compared to older studies, may be due to the children’s early STS knowledge. Several previous studies have shown a strong correlation between early sign language and literacy and spoken language (e.g., Svartholm, 2010; Hassanzadeh, 2012; Davidson et al., 2014) and it is likely that this also is the case for spelling knowledge. This suggestion is reinforced by the equal percentage of spelling errors in the deaf children who were the only group who had full STS knowledge since childhood in this, and in the previous study (Gärdenfors, 2016). An explanation may be that the majority of the children have deaf parents. Kelly (1995) and Humphries and MacDougall (1999) have documented that deaf adults are more prompt to use the *chaining*-method (showing a word or a sign and fingerspelling it to strengthen the link between the fingerspelling and the word) than the hearing adults – thanks to their own personal experience of learning to fingerspell twice (Padden, 2005). Using fingerspelling in Sweden is also reported by Bergman (2012) who observed that the adults use fingerspelling as a natural part of their communication with younger deaf children. When asked, many of the deaf parents of the participants of this study confirmed that they used fingerspelling to their children from an early age, saying that “fingerspelling is a crucial part of Swedish sign language.” Some of the parents had even read about the chaining-method and applied this on their children, since they believed that it would strengthen the relationship between fingerspelling and Swedish letters. The parents with STS knowledge may thus show how a word is spelled by fingerspelling it to their children, and in that way circumvent the sounding strategy by showing the visual alphabetic characteristics of a Swedish word to their CODAs and DHH-children. The understanding of the relationship between fingerspelling and how a word is spelled would therefore have been facilitated in children with STS knowledge, compared to the other children who had access to the sounding strategy only. This relationship is also in line with Padden and Ramsey (2000) who found a strong relationship between fingerspelling and reading ability.

In the next section, three spelling categories with patterns that were likely to be caused from sounding and visual strategies will be highlighted and discussed to deepen our understanding of the participants’ spelling.

## Aspects of Spelling Errors

The heterogeneous nature of the quantitative part of this study is complemented by a qualitative inspection of the spelling errors. The aim of the qualitative approach is to investigate some patterns relating to STS knowledge and hearing ability, as revealed by the quantitative part of the study. Below, both the similarities and differences will be presented. All the spelling results were based on misspelled words occurring during the process *and* in the final text, in order to show the relevant tendencies. In this section, patterns in length measures, the writing process, spelling in general, and the spelling categories of doubling errors, confusion of similar words and influence from STS will be discussed.

### Doubling Errors

An effect was found in doubling errors with the variable deafness (*p* = 0.07). The deaf children performed only 0.50% doubling errors compared to the others with 2.89%. We also observed that the many doubling errors in hard-of-hearing children were similar to the errors found in hearing children with typical errors such as “chokad” (‘shocked’), “öpen” (‘open’), “kunnde” (‘could’), instead of “chockad,” “open,” and “kunde”.

**FIGURE 2:**
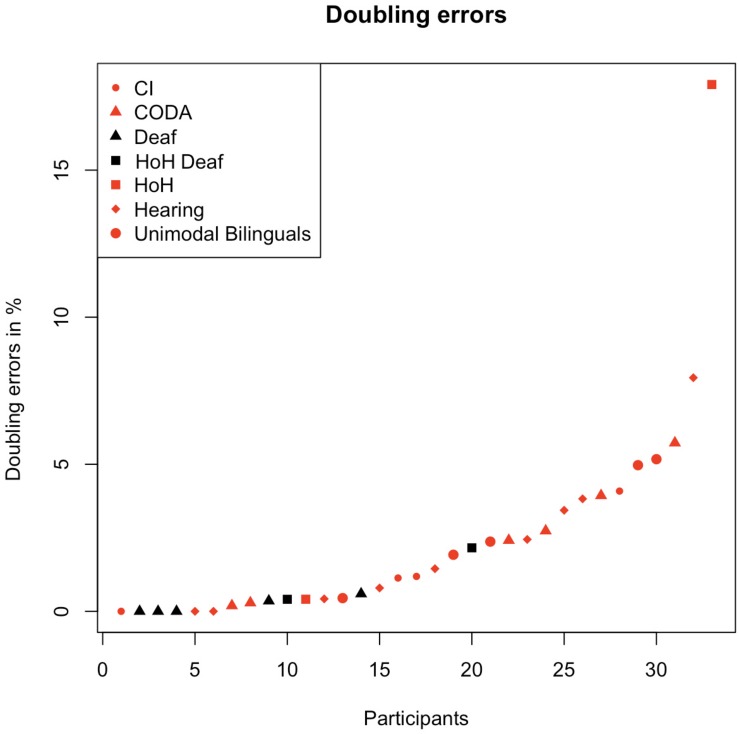
The distribution of doubling errors for children without deafness, that represent CI, CODA, HoH, hearing and unimodal bilinguals, all plotted in red. Doubling errors found in texts written by deaf children, or HoH deaf (children with residual hearing, but who have not developed spoken language) are plotted in black.

For the deaf children (black triangles in Figure 2), only two doubling errors were observed for five deaf individuals (representing an average of 0.4%), and the HoH and CI children showed on average 3.4% doubling errors, see Figure 2 for an illustration. However, two of them could not use their residual hearing in order to communicate by speech, and are therefore defined as “deaf” (plotted in black quadrats as HoH deaf). In those two individuals, only four doubling errors were identified, but those “doubling errors” seemed rather to have occurred by accident. Such an example was written as “dröme” instead of “drömmer” (‘dream,’ ‘dreaming’). Since the word lacks an “m*”*, it was counted as a doubling error following our criteria, however, this spelling also indicated a limited morphological knowledge. The Swedish noun is “dröm*”* (‘dream’), and the child was likely trying to use this form to create the verb, but did it incorrectly. Thus, this error was probably not caused by a sounding strategy. Taken together, the observations indicate that there is a relationship between deafness and lack of doubling errors, so one of the most important contributions here is that when a visual and a sounding strategy are available, the hearing, hard of hearing and children with CI seem to use the sounding strategy rather than the visual strategy.

### Confusion of Similar Words

A significant effect was found in the spelling category confusion of similar words and the predictor deafness. The deaf hearing children showed some patterns of non-semantic, however, visually similar looking words such as “fjälla*”* (‘scale’) instead of “fälla” (‘trap’), “brev” (‘letter’) instead of “bredde” (‘smeared’), “läder” (‘leather’) instead of “lägger” (‘lay’). The same phenomenon is also described by Wengelin (2002) and Gärdenfors (2016). One explanation may be that when a deaf child cannot confirm the spelling by sounding the letters, they will likely reach for the most salient letters of a word. Since the mental representation of the reached-for word may still be diffuse, a visually nearby word will be used instead. Since the children cannot confirm the meaning by sounding this out, the process will as a consequence result in a semantically incorrect word.

### Influence From STS

The last significant effect was found in the category of influence from STS and deafness. We identified three different kinds of influence from STS: by mouth; by handshapes; and by signs with different corresponding meanings in Swedish. First, mouth actions are a part of non-manual signals that are essential while signing because they fill important linguistic functions such as negation and adverbs for instance. Bergman (2012, p. 45, our translation from Swedish) writes: “[c]hildren acquire lexically determined mouth actions as natural, visual parts of the signs. Even before the age of two, such oral movements can be observed in children’s communication.” A similarity can be drawn with hearing children: when they learn new words, they also learn how to stress the vowels correctly. Since STS is the first language for some of the participants, we may expect that DHH participants, particularly those with no use of sound, rely on their acquisition of Swedish by looking at the mouthing (i.e., mouth actions based on borrowed elements from Swedish). However, length of mouthing is reduced to a few prominent segments (Bergman and Wallin, 2001), and we suggest that deaf children develop their phonological awareness on global characteristics, for example by how a word may look on the mouth (cf. Marschark, 2009) meaning that the DHH children rely on the spelling of the most prominent mouth segments, which is also reported by Sutcliffe et al. (1999). As a result, letters will be missing or reversed. This can be compared to how hearing children express words – a hearing child starts learning how to write words by uttering the word (Svensson, 1998), and can then be “misled” by the fact that some sound is missing in the spelling such as “hemst” instead of the correct “hemskt” (‘horrible’), in which the “k*”* is unpronounced.

(a) Deaf: “då gav katten henne (…) faskla”
(‘the cat gave her a (…) bottle’)

**FIGURE 3:**
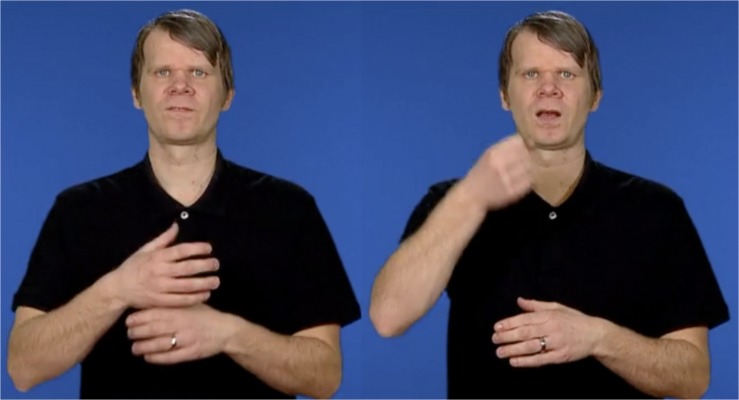
The supplied mouth movements while signing “FLASKA” are reduced to the most salient [FA] that result in a spelling error: “faskla” when the deaf relied on the mouth movement while spelling. The images come from https://teckensprakslexikon.su.se (The Swedish Sign Language Lexicon) and are used with permission of the copyright holder.

Example (a) represents an example of letter reversals that was likely caused by reduced mouthing in STS. The mouthing of the sign “FLASKA” (‘bottle’) is reduced to “FA” without any distinct movement for “L”, see Figure 3. Identical patterns of the Swedish word “flaska” have also been reported by Schönström (2010) and Gärdenfors (2016), who found several variants such as “falska,” “fasa” and “faka,” all started with “fa,” and not the supposed “fla” in deaf children’s written production.

(b) Deaf: “Mus blir så rätt när se (…)”
(‘The mouse becomes so right when it sees (…)’)

**FIGURE 4:**
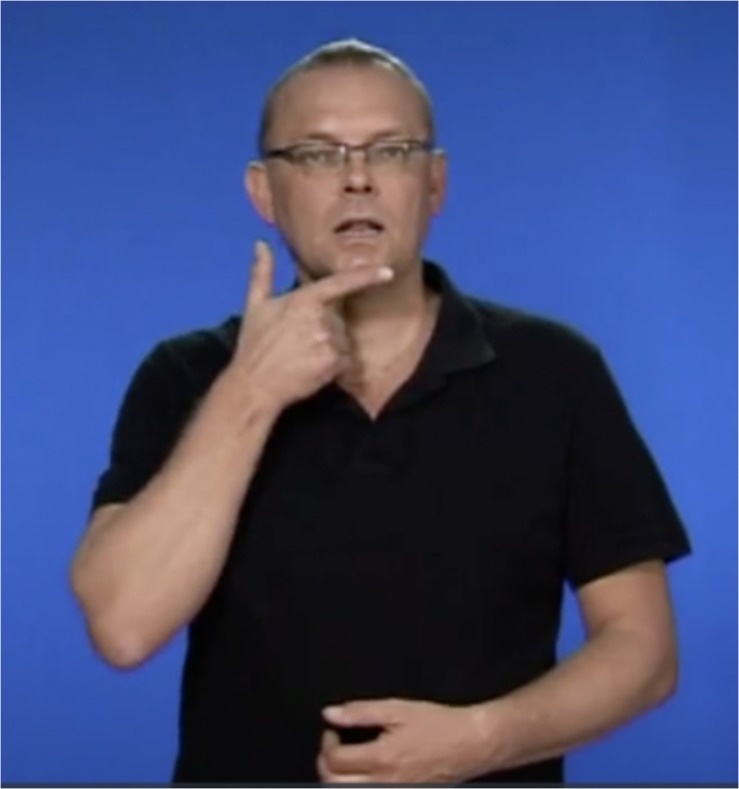
When a spelling error derives from a sign’s handshape. The picture shows the sign for “RÄDD” (‘scared’), and its handshape is formed as a “t”, resulting in the spelling error, “rätt” (‘right’). The image comes from https://teckensprakslexikon.su.se (The Swedish Sign Language Lexicon) and is used with permission of the copyright holder.

Second, example (b) shows when a profoundly deaf child bases a spelling on the handshape of a sign “as a false clue.” The word was supposed to be “rädd,” (‘scared’), but the word was written as “rätt” (‘right’). The STS handshape of the sign “RÄDD” is formed as a “t”, so there is a high probability that the child in writing replaced “tt” instead of the supposed “dd,” see Figure 4. Another interpretation is that this resulted from a confusion of similar words, since “rädd” and “rätt” are visually similar, and prior to not choosing a misspelled word of a particular category by neglecting another, this was also counted as a confusion of similar words.

(c) Deaf 1: “Lilly såg mus din fot*”*
(‘Lilly saw a mouse your foot (a mouse’s footprints)’)
(d) Deaf 2: “Och ser rosa katt din säng”
(‘The cat sees a sleeping mouse in your bed (his/her bed)’)
(e) Deaf 3: “Katt tog musen och går till ditt säng”
(‘The cat took the mouse and went to your bed (to his/her bed)’)

Lastly, examples (c), (d) and (e) show an overuse of the word “din/ditt*”* (‘your’) when the supposed words would be “hans*”* (‘his’) or the possessive affix *“*s*”*, in three profoundly deaf children, resulting in syntactical errors. The STS signs for “s”, “DIN,” “HENNES,” “HANS” and “DERAS” (‘your,’ ‘her,’ ‘his’ and ‘their’) are identical, representing a flat hand moving forward from the signer, and as a result, the children choose an incorrect Swedish word, with, however, the same underlying signs in STS. Example (c) indicates that the child did likely not know how to spell “musfotspår*”* (a mouse’s footprint) and tried to sign this word mentally from STS by rephrasing this to “mouse his/her foot”. Examples (d) and (e) are similar examples in which when the participants tried to express “his/her” by writing “din/ditt” which has an identical sign to “DIN.”

The findings of this study show both similarities and differences between the participants. The similarities could be found in the features of the writing process, particularly in words per minute, pause length in percentage, pauses per minute, pauses before and within words in percentages. Here, we may thus observer patterns typical for this age group. The differences are rather found in the variable deafness that explained the majority of the effects such as number of words, writing time, STS-test, doubling errors, confusion of similar looking words and influence from STS.

The first observation was that the bilinguals who were hard-of-hearing or CI-users showed a larger vocabulary than the bilingual deaf children. We suggest that it is due to the fact that they could acquire Swedish by means of spoken Swedish.

An essential contribution of this study is also that the hard-of-hearing and CI-users, despite their daily use of STS, seemed to rather rely on the sounding strategy than the visual strategy that was mirrored in recurrent doubling errors and letter substitutions, often caused by the sound. But their access to the visual strategy was not absent since their proportion of spelling errors were considerably lower compared to previous Swedish studies. This was explained as a facilitation from STS, from for example fingerspelling of their deaf parents who can demonstrate how a word is spelled through fingerspelling and circumvent the sounding strategy by showing the visual alphabetic characteristics of a Swedish word. Those visual strategies are reinforced, especially in deaf children, who showed a higher tendency to ‘spell as it looks,’ and in this have confused similar-looking words since they could not double-check the meaning by sounding it.

A final important finding was that the direct STS transfers (by mouth, by handshapes and by signs with different corresponding meanings in Swedish), could in the first instance be found in deaf children and not in the other children with STS knowledge. Since they did not have access to the sounding strategy, the visual strategy was the only one available. But, due to their limited vocabulary, and when the visual strategy was not available (i.e., due to drawing on their visual memory), they had to use other strategies – characteristics and signs from STS, such as direct translation from STS or spelling a word based on how it looks on the lips.

## Conclusion

Many of the spelling patterns found in this study confirm earlier findings in the field, that is, that a strategy that uses visual as well as auditory cues can, on the one hand, facilitate spelling, and on the other hand interfere with the spelling. Our present contribution is linked to how those strategies interact both together and separately. Our results indicate that auditory input is a crucial factor; when it is absent, the deaf children resort to visual strategies.

However, with regard to the DHH children, it is difficult to isolate and investigate the impact of auditory and visual input respectively. This needs to be addressed in future studies. Nevertheless, our results indicate that DHH children benefit from using input from both modalities. Further, the results have pedagogical implications and demonstrate the importance of teachers’ awareness of the special challenges in learning to spell that the groups of STS and DHH children face. The absence of auditory input calls for an early and continuous input of visual channels, such as exposure to written words through reading, and by explicit training in the relationship between written words and fingerspelling. The latter point has also been shown to be beneficial even for children with residual hearing and also for hearing children as a complement to the auditory strategy.

According to SOU (2016:46) (an official investigation of the Swedish government), the majority of the congenitally deaf Swedish children receive CI before the age of 8–9 months, and some will receive CI as early as 5 months. If the children receive it before the age of 9 months, it is likely that many of them will develop an adequate spoken language. This investigation also reports that 80–90% of those Swedish children with CI attend a mainstream school, and the remainder who do not, attend special schools because of hearing problems or intellectual delays. This study on how sign language relates to spelling makes a significant contribution to the understanding of how basic writing skills are established in this group. Since the children with STS knowledge in the present study showed considerably fewer spelling errors compared to earlier studies, we want to highlight the supporting role that sign language seems to have in developing spelling skills. Having access to a bilingual repertoire with auditory as well as visual input provides these children with a wider range of strategies to make use of for spelling.

## Data Availability Statement

The datasets generated for this study are available on request to the corresponding author.

## Ethics Statement

The studies involving human participants were reviewed and approved by Etikprövningsnämnden in Stockholm. Written informed consent to participate in this study was provided by the participants’ legal guardian/next of kin. Written informed consent was obtained from the individual(s), and minor(s)’ legal guardian/next of kin, for the publication of any potentially identifiable images or data included in this manuscript.

## Author Contributions

MG (first author) mainly responsible for the data collection and analysis, dissemination of the results, and manuscript writing. VJ (supervisor) and KS (head supervisor) involved in writing parts of the manuscript.

## Conflict of Interest

The authors declare that the research was conducted in the absence of any commercial or financial relationships that could be construed as a potential conflict of interest. The handling Editor declared a shared affiliation, though no other collaboration, with one of the authors VJ at the time of the review.

## Footnotes

1 The majority of the students in Swedish schools start learning English in the 3–4th class and may have basic knowledge of English by the age of 10–11.
